# Corrosion Prediction of Weathered Galvanised Structures Using Machine Learning Techniques

**DOI:** 10.3390/ma14143906

**Published:** 2021-07-13

**Authors:** Marta Terrados-Cristos, Francisco Ortega-Fernández, Guillermo Alonso-Iglesias, Marina Díaz-Piloneta, Ana Fernández-Iglesias

**Affiliations:** Project Engineering Department, University of Oviedo, 33004 Oviedo, Spain; fdeasis@uniovi.es (F.O.-F.); guillermo.alonso@api.uniovi.es (G.A.-I.); marina.diaz@api.uniovi.es (M.D.-P.); fernandeziana@uniovi.es (A.F.-I.)

**Keywords:** weathered galvanised steel, corrosion, predictive models, optimisation

## Abstract

Galvanised steel atmospheric corrosion is a complex multifactorial phenomenon that globally affects many structures, equipment, and sectors. Moreover, the International Organization of Standardization (ISO) standards require specific pollutant depositions values for any atmosphere classification or corrosion loss prediction result. The aim of this research is to develop predictive models to estimate corrosion loss based on easily worldwide available parameters. Experimental data from internationally validated studies were used for the data mining process, basing their characterisation on seven globally accessible qualitative and quantitative variables. Self-Organising Maps including both supervised and unsupervised layers were used to predict first-year corrosion loss, its corrosivity categories, and an uncertainty range. Additionally, a formula optimised with Newton’s method has been proposed for extrapolating these results to long-term results. The predictions obtained were compared with real values using Euclidean distances to know its similarity degree, offering high prediction performance. Specifically, evaluation results showed an average saving of up to 16% in coatings using these predictions. Therefore, using the proposed models reduces the uncertainty of the final structures state by predicting their material loss, avoiding initial over-dimensioning of structures, and meeting the principles of efficiency and sustainability, thus reducing costs.

## 1. Introduction

Multiple metallic structures and equipment operate in outdoor conditions [1]. In such cases, one of the main problems related to their stability and durability is corrosion [2,3]. World Corrosion Organization (WCO) estimates the world direct cost of corrosion to be between 1.3 and 1.4 trillion EUR, 3.1% to 3.5% of a nation’s GDP annually [4].

Corrosion is a very complex phenomenon based on the degradation of a material or its properties due to its reaction with the environment [5]. Multiple factors [6], particles [7], and variables [8,9] are involved. The character of the attack and the corrosion rate are consequences of the system formed by metallic materials, atmospheric environment, technical parameters, and operating conditions [10]. Corrective factors are introduced in the design phases to guarantee the structure’s integrity during its useful life [11]. However, the difficulty of quantifying the material loss causes unnecessary over-dimensioning, leading to superfluous costs and resources consumption [12]. Proper management of this complex multifactorial phenomenon is key to sustainable development [13].

To ensure the integrity of the outer layer, structures are designed with physical protection. Historically, metallic zinc has provided excellent corrosion protection of steel structures [14]. Unfortunately, corrosion damage also occurs in such systems [15]. Since corrosion leads to a mass loss, an excess thickness is often considered to ensure service life. This not only increases manufacturing cost but also does not satisfy the principles of sustainable engineering efficiency [16]. Therefore, lacking an automated monitoring system or predictive model, routine thickness monitoring would be required [17]. These phenomena have drawn increasing attention in recent decades due to the resulting catastrophic accidents [18] and the growing demand for sustainable designs [19]. For an optimal selection of materials, atmospheric aggressiveness must be considered. Depending on this, coating needs can be set.

The current regulation regarding galvanised metallic structures (ISO 9223:2012 [20]) groups the corrosivity level of an atmosphere into six categories. After studying the effect of corrosion on standard samples during 1 year of weathering exposure, the level of corrosion rates achieved can be established by measuring weight losses for different materials. This material’s loss due to corrosion is commonly used as an initial measure for determining coating requirements. However, material loss margins are allowed within these categories, and coating thickness designs based on them are not fixed. These margins imply variability in the amount of material that can be translated into increased costs.

According to [20], two methods are proposed to classify the corrosivity of atmospheric environments, depending on the availability of experimental data. When experimental data are available, dose–response functions can be used. However, when no experimental data are available, corrosivity category estimation using the informative procedure is recommended, and as stated in the norm, it is based on the comparison of local environmental conditions with the description of typical atmospheric environments, which may cause misinterpretations [21]. Finding the optimum point between efficiency and competitive price, while remaining within limits, is therefore challenging given the lack of characterisation of the specific construction site.

The objective of this work is to develop machine learning models that, by analysing real cases, predict corrosion mass loss of zinc coatings over time. The aim is to characterise an environment without requiring long testing periods and sampling and generalising it to any location worldwide, with the data available from international studies. This considerably increases the existing knowledge about coated steel structure corrosion and extends it to the full diversity of atmospheres, thereby reducing the uncertainty of its final state.

This paper starts with a state-of-the-art analysis. Then, it explains the creation of the database through the characterisation of each sample. Next, the applied methodology is explained, and modelling and evaluation techniques are defined. Finally, results are discussed, and the conclusions obtained in this research are proposed.

## 2. Literature Review

There is a wide range of corrosion problems in the industry, resulting from the different combinations of materials, environments, and service conditions [22]. Therefore, the concern about corrosion is not new. The science of atmospheric corrosion started with Faraday in the nineteenth century [23]. Another important contribution was made by Vernon who began systematic experiments in atmospheric corrosion in the 1920s [24]. In 1986, Benarie and Lipfert published their work on atmospheric corrosion [25], relating this phenomenon to the concentration of certain pollutants and pH of the rain. Subsequently, Feliu et al. developed regression equations for mild steel, zinc, copper, and aluminium [26].

There are several kinetic corrosion models that attempt to predict atmospheric corrosion over time: the general linear model [27], the power function models [28], and the power-linear models [29]. However, the corrosion process is influenced by multiple environmental factors [30]. Therefore, these corrosion kinetic models are valid at specific locations. When the environmental condition changes, the model may no longer be applicable [31]. It would be interesting to classify the aggressiveness of different atmospheres, which would allow preventive measures to be taken. Therefore, it is important to introduce the interaction parameters between environmental factors and corrosion rates for their efficient prediction.

In accordance with this approach, the ISOCORRAG program was launched in 1986 [32]. The ISO 156 technical committee developed this project with the intention of obtaining sufficient information to standardise atmospheric corrosion on metals and alloys. Four international standards were created as a result of this project: ISO 9223 [21], ISO 9224 [33], ISO 9225 [20], and ISO 9226 [34]. Since then, these standards have served as practical guidelines and aids for the design of both structures and their corrosion protection. In September 1987, the Executive Body for the Convention on Long-Range Transboundary Air Pollution (CLRTAP) decided to launch an International Cooperation Program with the United Nations European Economic Commission (ICP/UNECE) [35] whose objective was to carry out a quantitative assessment of the effect of pollutants on atmospheric corrosion [6]. In addition, a third cooperative program was launched, named MICAT [36] (Ibero-American Atmospheric Corrosivity Map). Its objective was to understand the mechanisms that take place when this phenomenon occurs, to generate, with the data obtained, mathematical models to calculate corrosion as a function of climate condition or pollutant levels [13]. The three projects evaluated corrosion by measuring mass loss and were based on what was indicated in the standard for measuring SO_2_ or Cl^−^ levels and other pollutant concentrations.

In 1992, the ASTM (American Society for Testing and Materials) published a study discussing an alternative method for measuring corrosion penetration, with models that are tighter and more rational than the traditional potential model [37]. In 2003, several workers compiled atmospheric exposure data from many research reports and journal articles [38]. R.E. Melchers, an engineer at Newcastle University, focused on studying the corrosion of metals in marine atmospheres in his studies in 2008 [39] and 2013 [40]. Later, Morcillo et al. [27] made a comprehensive compilation in the scientific literature on weathering steel atmospheric corrosion [6]. In addition, they developed Damage Functions to know the damage that a metallic structure can suffer depending on weathering conditions. In the subsequent years, there have been local experimental studies to characterise this phenomenon, such as those in Greece [41] and the Czech Republic [42].

The dose–response function is the most widely used. It directly correlates the influencing environmental factors with the corrosion parameters [43]. The basic form of this function follows the simple linear [36,44] or logarithmic–linear relationships [45]. However, many researchers also started to depart from judging the effect of each environmental factor separately and established a new multi-factor combination model [46,47]. A response surface model (RSM) takes into account the interactive effect and the non-linearity of the atmospheric corrosion process and allows a better approximation compared to conventional dose–response function models [48]. The models offer a closer approximation of corrosion rate by introducing different input variables. Temperature, humidity, sulphur dioxide concentration, and chloride concentration are typically used.

In conclusion, there are different options to predict corrosion rates of metals based on experimental input data. However, for the cases when pollutants’ concentration is unknown, the options are limited. Time and cost constraints make the development of these measurements difficult as they would be unrepresentative when only completed at a specific point in time. As the environmental conditions continuously change, it is necessary to know their distribution over larger distances and longer periods of time. All corrosion related research carried out so far showed that there are certain factors that clearly influence the corrosion process. Regarding atmospheric corrosion, the factors include temperature, relative humidity, precipitation level, and pollutant concentrations (SO_x_, Cl^−^, etc.) [49,50]. A combination of parameters, such as Time of Wetness (TOW), is also used. TOW represents the fraction of time when relative humidity exceeds 80% and ambient temperature is above 0 °C (h/year) [51].

Climate has a significant influence on corrosion since some of the factors mentioned above depend on the climatic zone. A Köppen–Geiger classification [52] is the most popular technique for climate characterisation. According to this method, six precipitation levels can be distinguished [52]: desert (0), steppe (1), totally humid (2), summer dry (3), winter dry (4), and monsoon (5). Temperature and relative humidity are easily analysable climatic variables, and their values are generally accessible. There are also additional factors besides climate, mainly derived from human activities, whose importance is also significant. It is evident that the most populated and most-developed areas with accumulations of vehicles and high industrial activity have greater corrosive potential. It is also known that materials situated in areas closer to the sea tend to have a worse corrosion performance. Therefore, it is necessary to include these additional factors as well as they are critical for the successful operation of the model.

## 3. Materials and Methods

### 3.1. Data

**Figure 1:**
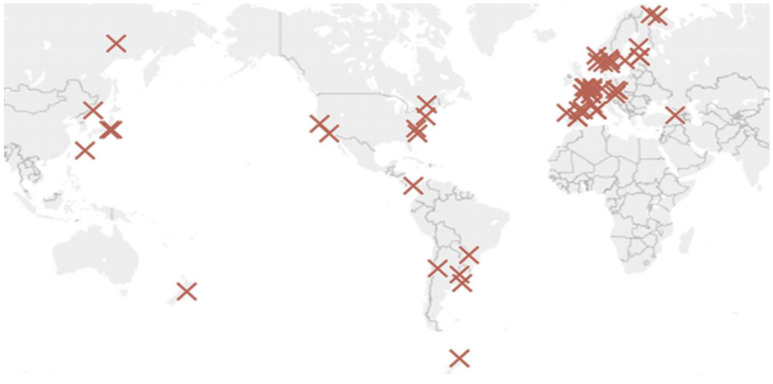
ISOCORRAG program sample’s location.

This work seeks a more practical approach to characterise the environment. After a complete analysis of the data from existing experimental studies, it has been concluded that ISOCORRAG program data [32] should be used as it also analysed the corrosion in helical samples. Corrosion rates on helical samples have higher average corrosion rate values and do not limit corrosion loss to a single direction. This approach is useful in our case, as it more closely relates to galvanised structures used in civil engineering. Besides, it includes enough helical specimens distributed globally to represent a wide variety of cases. The project was carried out at more than 50 different locations in Asia, Europe, and America (Figure 1). During the ISOCORRAG program, the exposed specimens were used to determine the first-year corrosion rate. Nevertheless, some of the specimens were also used to study long term corrosion exposure. Grouped in different sets, triplicate samples were exposed every 6 months, and left for up to 1 year. The monitoring process lasted from 1986 to mid-1989.

ISO 9223 and ISO 9224 standards are highlighted for this project. First, ISO 9223:2012 [20] divides the corrosivity of atmospheres into 6 categories. Each of these categories corresponds to a different corrosion level. For zinc, data are shown in Table 1.

Second, ISO 9224:2012 proposes a relationship for long-term corrosion exposures. This relationship is based on the power function according to the following equation:

(1) $$D=r_{\mathrm{corr}}\;t^{b}$$

In Equation (1), r_corr_ is the first-year corrosion rate, *t* is the number of years to be analysed, and *b* is the environment and metal-specific time exponent.

#### 3.1.1. Variables

Willing to characterise any location worldwide, its atmospheric corrosivity and climate need to be considered. For this work, three specific types of atmospheric environments have been introduced as binary synthetic variables, trying to represent the behaviour of sulphates-related pollution and chlorides deposition:

- Industrial/Non-industrial: industrial are areas with fossil fuel combustion industries (refineries, thermal power plants, etc.).
- Marine/Non-marine: this characterisation has been made according to the distance from the coast, considering as Marine any location within 15 km from the seashore [53,54].
- Urban/Rural: locations with more than 5000 inhabitants or 300 inhabitants per square kilometre have been considered urban locations [55].

**Figure 2:**
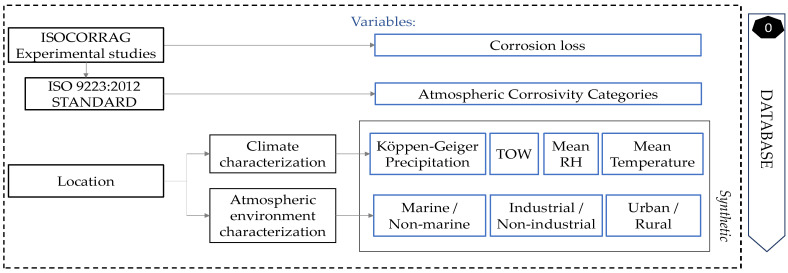
Flow chart for database creation and future locations characterisation.

Regarding the climate characterisation, temperature, relative humidity, TOW, and Köppen–Geiger level of precipitation were the main characteristics, unified in a simple, accessible, and complete way. Therefore, a total of seven numeric predictor variables were set for the model: mean annual temperature, mean annual relative humidity, TOW, precipitation, industrial, marine, and urban. The variable to be predicted was the zinc corrosion loss during first-year exposure, directly taken from experimental studies, and its atmospheric corrosivity category, based on the standard. Each sample was characterised, following the rules mentioned above, as explained in Figure 2.

A summary of variables is shown in Table 2. The mean annual temperature is represented as T_annual and mean annual relative humidity as RH_annual in the table.

#### 3.1.2. Data Analysis

**Figure 3:**
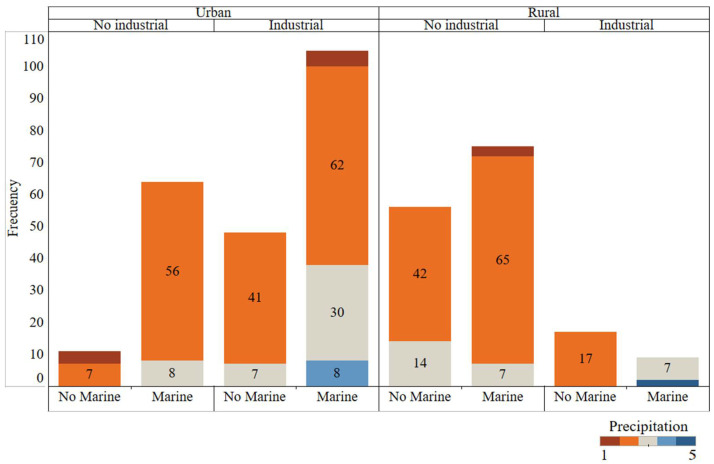
Frequency graphical analysis of the categorical variables. All possible atmospheric environment combinations are represented and coloured by precipitation type.

Data quality and representativeness are crucial for modelling; otherwise, the results obtained would be inconsistent. Frequency distributions of the 4 discrete variables are shown in Figure 3. All possible combinations between different environment types (Rural/Urban, Industrial, Marine) have been observed. In addition, colours show the number of samples in each of the 5 possible precipitation levels. All precipitation levels were represented; however, there some combinations were represented more often than others (urban, industrial, and marine zone).

**Figure 4:**
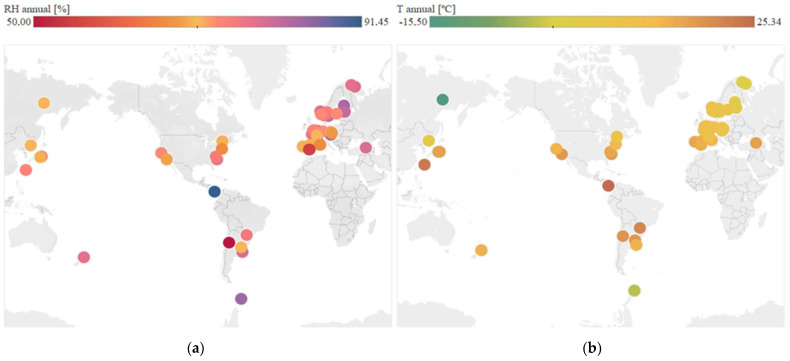
Analysis of continuous variables at each location. (**a**) Distribution of mean annual relative humidity. (**b**) Distribution of mean annual temperature.

Regarding continuous variables, Figure 4 shows the geographical distribution of temperature and mean annual relative humidity in each location, according to the numerical values obtained. The data are obtained from web services that use weather stations spread all over the world. Worldwide distribution of cases has been achieved.

### 3.2. Methodology

**Figure 5:**
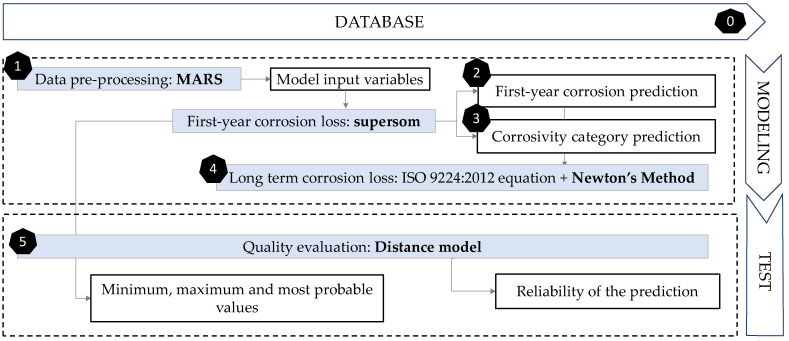
Flow chart showing the methodology followed in this paper. The six phases proposed are exposed as shown.

The methodology followed in this paper consisted of 6 phases (Figure 5). The preparatory stage (stage zero) in the previous subsection was concluded with the creation of the database. Then, the remaining five phases included modelling and testing. The first step for data pre-processing was to identify input variable’s importance for better understanding their behaviour and obtaining additional information regarding their usefulness in the final model. This was completed using Multivariate Adaptive Regression Splines (MARS, Step 1). Then, the next phase was to define the first-year corrosion loss of galvanised steel. Self-Organising Maps (SOM) were used, including various layers (supersom) of both supervised and unsupervised learning. The next two steps used the result of the various layers of this algorithm. The first layer has been the result of using unsupervised SOM, according to the relationships between the 7 main variables. Zinc corrosion loss during first year of exposure (Corr_Zn, in µm) was the output variable to be predicted (Step 2). 

The advantage of SOM maps is that in addition to assigning an individual value, an uncertainty range is also given, obtained by adding the minimum and maximum value within each neuron. Besides, it is intended that in addition to self-organising according to the input variables, supersom networks group the data according to the various corrosivity categories. Then, the second one of the two output layers would be the result of organising corrosion in a supervised output layer that will assign the corresponding ‘corrosivity category’ value set to each node by the standard (Step 3). Furthermore, the corrosivity is not constant with respect to exposure time. In most cases, it decreases with increasing exposure due to accumulation of corrosion products on the surface. Step 4 includes optimising the formula that allows the extrapolation of these results to long term results. With Newton’s method, a nonlinear regression of the formula used by ISO 9224 (Equation (1)) was performed to optimise the value of variable *b*.

Finally, to test the quality of the predictions, a model based on Euclidean distances was used (Step 5). This model analyses the model input variables, trying to find the most similar cases in the database to show their corrosion value and its similarity degree (quality). Then, in this fifth phase, the results obtained were compared with existing real cases to measure the quality of predictions using a Euclidean distance model. Although both supersom and distance models start from the same database and have the same inputs, their purposes are different. While supersom model gives a corrosion prediction, and a corrosivity category, the distance model sets the quality of that prediction.

#### Techniques

- Multivariate Adaptive Regression Splines (MARS)

One of the most widely used algorithms for solving adaptive computing problems is MARS [56]. This method consists of approximating an unknown function by the linear combination of a set of basic functions (products of the model variables) [57]. Among the key points of the algorithm, it stands out that it autonomously selects the relevant variables and interactions between them for each subregion. Thus, the dimensionality reduction of the problem is performed directly by the model, with the advantage of being locally carried out. Precisely, this benefit can be used to analyse the relevance of the variables likely to subsequently participate in the model.

- Self-Organising Maps (SOM)

The clustering model, known as SOM, is an unsupervised Artificial Neural Network (ANN) presented in 1982 by T. Kohonen [58]. This model is based on certain evidence discovered at brain level and performs a reduction of the dimensionality of the input space to produce topologically ordered maps. This type of network has competitive, unsupervised learning. The network itself is in charge of self-organising and discovering common features, regularities, correlations, or categories in the input data [59,60].

**Figure 6:**
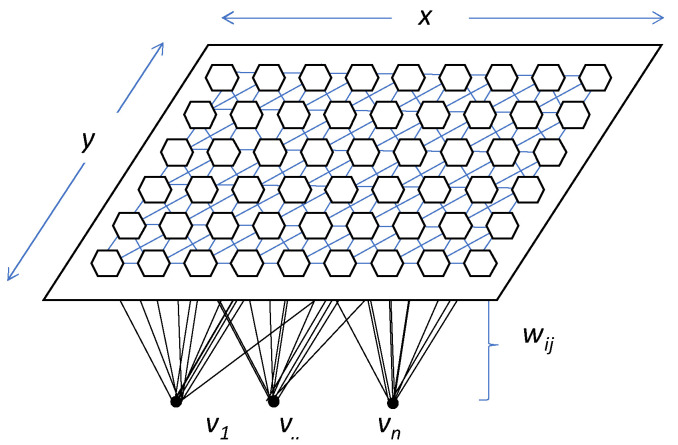
General example of SOM model’s topography. Dimensions are expressed by *x* and *y*; *v*_1–n_ represent each one of the input neurons, and *w*_ij_ is the weight of each vector according to Kohonen’s notation.

Figure 6 shows the architecture of the model and how each input neuron is connected to one of the output neurons by weights (*w*, according to Kohonen’s notation). The output neurons will therefore have an associated vector of weights which is called the reference vector (or codebook), also constituting the average vector of the category represented by the output neuron [61,62].

SOM’s utility lies in the holistic visual interpretation of the output rather than in understanding the underlying processes [63]. Roughly speaking, the output layer (i.e., the self-organising map itself) contains neurons organised in a rectangular or hexagonal lattice to represent the entire dataset [58].

The goal of this learning is to categorise the data fed into the network. Similar values are classified into the same category and, therefore, should activate the same output neuron. Since this is an unsupervised method, classes or categories must be created by the network itself through correlations between the input data [64]. However, SOM can also be used for pattern recognition (supervised learning). The information is given at the end of the training: if classification is involved, as in this case, the winner-takes-all strategy is used. This principle can be extended to more layers, generating super-organised maps (supersom). For each layer, a similarity level is calculated, and the individual similarities are combined into a single value which is used to determine the winner node.

- Newton’s method

This nonlinear regression uses Newton’s Surface gradients, which is an unconstrained linear regression method based on that gradient. The gradient information is provided by analytically computed gradients. Design variables are modified, while their impact on the objective function is analysed [65].

- Euclidean distance model

The operation of this model is based on Euclidean distances ($d_{E}$). This is a non-negative function used to calculate the distance between two points P = ($p_{1};{\;p}_{2};\ldots ;{\;p}_{n}$) and Q = ($q_{1};{\;q}_{2};\ldots ;{\;q}_{n}$) on an n-dimensional space [66]. It works on the basis of the Pythagoras Theorem (Equation (2)) [67]. Results evaluation using this method involves checking that the model gives a 100 % quality in all the cases studied, i.e., that it perfectly finds its counterpart.

(2) $$d_{E}\left(P,Q\right)=\sqrt{{\left(p_{1}-q_{1}\right)}^{2}+\cdots +{\left(p_{n}-q_{n}\right)}^{2}}=\sqrt{\overset{n}{\underset{i=1}{\sum }}{\left(p_{i}-q_{i}\right)}^{2}}$$

To summarise, Table 3 shows the different algorithms used in each phase of the data mining process.

## 4. Results and Discussion

Results obtained in each of the phases are presented below.

### 4.1. Data Pre-Processing Using MARS

**Figure 7:**
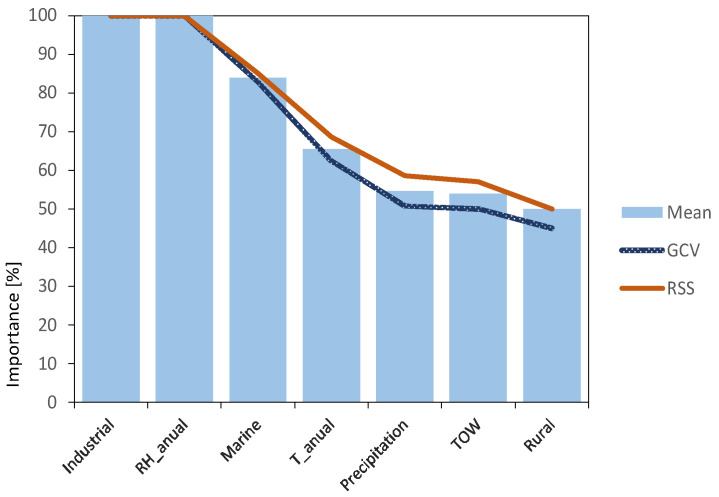
Variable importance analysis results, using MARS algorithm.

The importance of each of the variables has been analysed, assessing their influence on the variable to be predicted. Two statistics were used: generalised cross-validation criterion (GCV) and residual sum of squares (RSS). Both criteria results (blue and red lines) together with the mean of both results (light blue bars) are shown in Figure 7.

It is clearly evidenced that variables related to atmospheric pollutants SO_2_ (Industrial) and Cl^−^ (Marine) are the most important factors, together with relative humidity, in agreement with what was previously described in the literature review. They can all be considered as independent variables, susceptible to providing the model with enough information to obtain valuable predictions.

### 4.2. First-Year Corrosion Prediction

**Figure 8:**
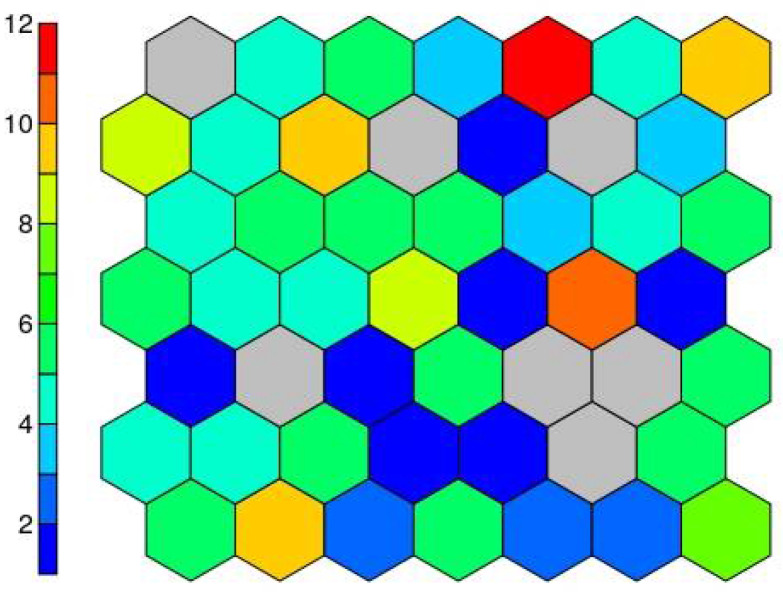
Number of cases on each neuron.

The result of the supersom model is a mesh of 7 × 7 hexagonal neurons trained with the Kohonen algorithm, which provides a good representation of the sample space. The resulting trained map contains all the data in a vector structure so that the training data falls on each of the neurons (Figure 8).

**Figure 9:**
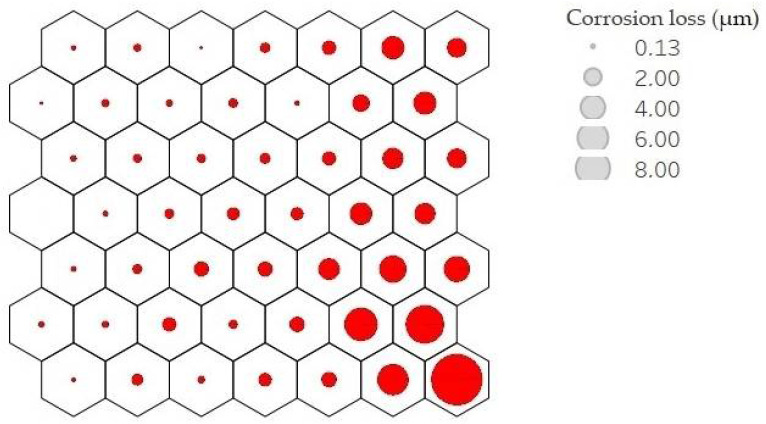
Mean corrosion values per neuron. Corrosion loss in µm per year is represented by circle size.

Each neuron, filled or not, is represented by a codebook. These neurons are arranged in such a way that nearby neurons represent points closer to each other. Analysing the result of the average corrosion values per neuron along the mesh, it can be clearly seen how the mesh is growing towards the lower right corner. Figure 9 shows this result; the larger the circle size, the higher the average corrosion. Keeping the neighbourhood properties, a uniform behaviour is shown, which indicates good training results. 

### 4.3. Corrosivity Category Classification

**Figure 10:**
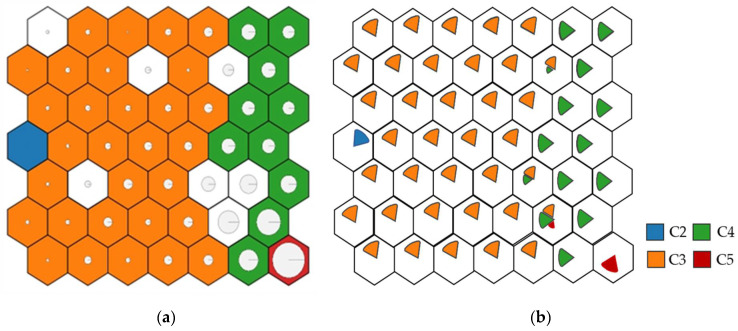
Corrosion zones according to the environment. (**a**) Corrosion representation (larger circle, more corrosion). (**b**) Corrosivity category representation, according to ISO 9223:2012 standard.

When analysing the results of both output layers, represented in each neuron by its corrosion rate value, the neurons were grouped, forming zones mostly corresponding to one type of atmosphere (Table 1). The zones division with different corrosion rates is given in Figure 10. Both C1 and CX categories were filtered out of the dataset due to a lack of consistent data. Thus, the far-left zone corresponds to C2 atmospheres, the left zones to C3, the right zones to C4, and finally, the lower-right end to C5. There is also a transition between the values so that the C5 are in contact with C4, C4 with C3, etc., demonstrating an optimal training.

**Figure 11:**
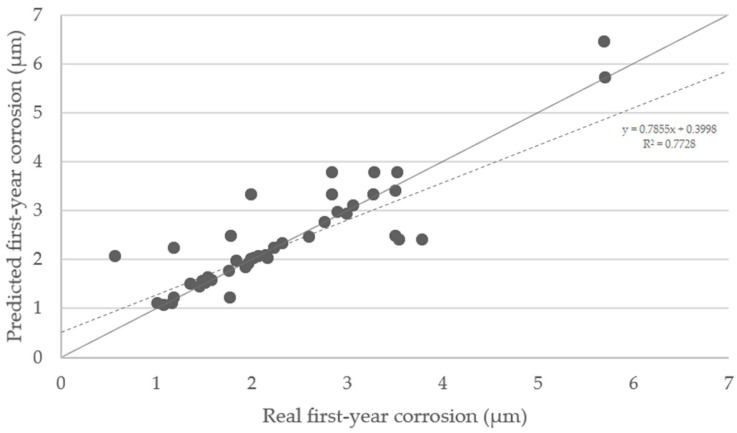
Predicted first-year corrosion values in micron vs. real first-year corrosion values. The dashed line is the regression line (R^2^ = 0.7728). The points situated on the diagonal grey line represent an optimal training.

The predicted first-year corrosion rates using SOM trained network were compared with real values. A satisfactory correlation has been obtained (Figure 11), although not all points perfectly matched their counterpoints. The ideal situation would be if the predicted values all lied on the diagonal line. The points tend to be located on the upper side of the graph, meaning that predictions are conservative, and the decisions made based on them can provide greater safety.

From the trained network, it is possible to determine the corrosion rate of any situation to be studied. When introducing a new case to the model, it finds the node that most closely resembles its input variables. Thus, the output of the model is the corrosion rate of that node. The uncertainty range is also given, including the minimum and maximum values within each neuron. This can be seen with the following example for a case with the characteristics defined in Table 4.

**Figure 12:**
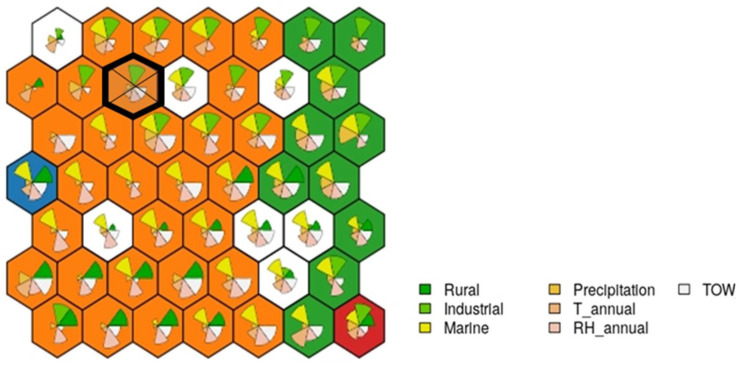
Case study example: the cake portions shown at each node show the contribution of each variable; the larger the size, the greater its final weight.

The case falls into the neuron indicated in Figure 12, which consists of 10 examples.

Table 5 shows all results obtained. Different conclusions can be made by selecting the maximum (Corr_max), minimum (Corr_min), and average (Corr_avg) values of the examples in one single neuron. As a result, when the values with the most or least corrosion occurring within the projects in the neuron are chosen, the optimistic and pessimistic predictions can be obtained. Alternatively, β-distribution is used to determine the ‘most probable’ rate of Corr_Zn, using the maximum, minimum, and average values. On the other hand, the category is awarded by the weighted average of the categories in each case. In this case, since all cases are C3, C3 is its category.

**Figure 13:**
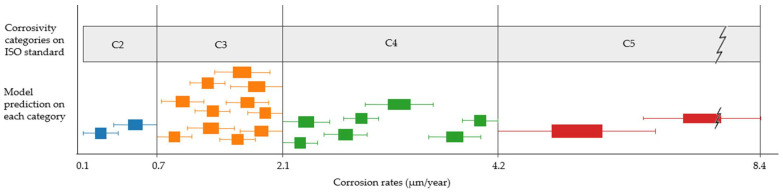
Comparison between each category range offered by the standard using the informative procedure and the possible mean values and uncertainties offered by the model, represented by clustered boxplots on each category.

Comparing the range given by the model with the range given by the existing standard, it is observed that the latter represents a much higher uncertainty for each corrosivity category. Extending this comparison to the entire study scope, possible model predictions for each category, clustered on similar values and represented by boxplots, can be presented (Figure 13). Although not all categories are equally distributed, they show, in general, narrower intervals.

This study is presented as a possible alternative to the informative procedure of the ISO standard when there is no experimental data available. The results of the informative procedure regarding atmospheric categorisation provide a range of mass losses for each material. The current trend among companies and engineers, when no specific experimental information is available, is to use the highest value of each category to make their decisions. Since corrosion loss values are directly related to the required coating thickness, the higher the corrosion loss value, the more coating is required. A coating thickness can thus be directly determined by the predicted material’s loss.

The material requirement for coatings can be compared with the largest measurement proposed by the standard in each category and with the value predicted by the model. Following the example above, when using a Zn-coating of 1.6 µm (Corr_avg) instead of 2.1 µm (maximum in the range given by ISO), a 24% reduction in material’s costs is obtained. It is then proposed to carry out this comparison for the rest of the points studied. From a more conservative perspective, comparing the maximum predicted value (Corr_max) with the maximum proposed by the standard using the informative method can also be used. In this way, uncertainties are also considered. By performing this for all data studied during the evaluation phase, an average saving of 16% in coating material is obtained.

### 4.4. Long-Term Corrosion Prediction

Once the first-year corrosion rate provided by the supersom model is known, the long-term loss can be identified thanks to the optimised Equation (1). Table 6 shows the different values obtained by this optimisation method for each of the corrosivity categories.

**Figure 14:**
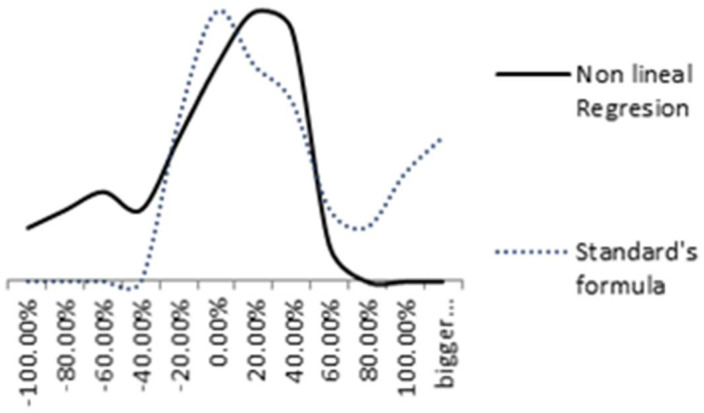
Comparison between Nonlinear regression and standard’s formula relative errors.

Figure 14 compares the distribution of relative errors of both models. The nonlinear regression relative error is represented by a solid black line and the standard formula’s relative error (ISO 9224) by a blue dashed line. A more uniform distribution is achieved in the nonlinear regression model.

### 4.5. Quality Evaluation

For the correct functioning of the model, data were normalised. According to the previous criteria, the most similar options are shown. The best way to show the results of this last model is using an application example, which is presented in Table 7. The quality row shows the percentage assessing the prediction’s quality. The first column represents all input values of the example. The next three columns show the most similar real results in the database. 

Results obtained above show high prediction reliability. Cases similar to the one under study have been found in the database. The model could also give a satisfactory result for a case that is not included in the database. Ideally, the results obtained with the proposed methodology should be compared with the results obtained with existing methods in the literature. However, since the innovative premise of this study is based on adapting the input variables to avoid the need for pollutant-specific data, such a comparison cannot be made. One of the differentiating factors of this classifier model is that to obtain a corrosion loss rate, values for pollutant concentrations are not needed. Consequently, it may be concluded that the different algorithms developed are a good alternative for technicians and engineers to make informed decisions based on their level of risk acceptance. To sum up, given a specific location and based on the available data, these models can determine the Zn-coating thickness needed for a successful short- and long-term corrosion resistance, providing the most probable, optimistic, and pessimistic predictions.

## 5. Conclusions

In the present work, various models for predicting galvanised coated steel corrosion damage of metal structures exposed to weathering have been developed. The following conclusions can be drawn from this research.

The application of a supersom algorithm is considered for first-year corrosion prediction, which allows categorising any environment while obtaining a predicted value, with satisfactory results. In the cases when no experimental data are available, the model can be an alternative to the conventional informative method based on pollutant input variables. The model presented in this work could help civil engineering companies to optimise the ratio between the minimum coating required and maximum service life, thus contributing to a significant lifetime extension of steel structures.

The main limitation of the model is that it lacks statistical metrics to evaluate the performance. To solve this and explore the performance and quality of the predictions, a quality model based on Euclidean distances was proposed. A long-term corrosion prediction was also optimised based on standards ISO 9224:2012 formula and the exponential coefficient with Newton’s method.

To cover all different atmospheric environments, more specific characterisations are required. The future research will focus on including the development of physical variables, such as wind speed and wind direction. It is also important to feed the model with more examples from the lesser-represented categories, as there are notable differences between C3/C4 categories and the remainder of the cases. Adding new metallic materials will also be explored, following the same methodology, possibly leading to the development of new prediction models.

## Figures and Tables

**Table 1:** Corrosion rates of zinc for first-year exposure for different corrosivity categories according to ISO 9223:2012.

Corrosivity Category	Corrosivity	Unit	Zinc
C1	Very low	µm/year	r_corr_ ≤ 0.1
C2	Low	µm/year	0.1 < r_corr_ ≤ 0.7
C3	Medium	µm/year	0.7 < r_corr_ ≤ 2.1
C4	High	µm/year	2.1 < r_corr_ ≤ 4.2
C5	Very high	µm/year	4.2 < r_corr_ ≤ 8.4
CX	Extreme	µm/year	8.4 < r_corr_ ≤ 25

**Table 2:** Information on new continuous and discrete variables added.

Continuous Variables					Discrete Variables	
Variable	Unit	Min	Avg	Max	Variable	Range
T_annual	°C	−15	14.5	29.1	Marine	0 (Non-Marine)–1(Marine)
RH_annual	%	33	74.7	98	Industrial	0 (Non-industrial)–1(Industrial)
TOW	h/year	37	2723	6350	Rural	0 (Urban)–1(Rural)
					Precipitation	1–5

**Table 3:** Summary of all models used.

Phase	Algorithm
Pre-processing data	MARS
Modelling	
Corrosivity category prediction	superSOM
First-year corrosion prediction	superSOM
Long-term corrosion prediction	Newton method
Quality evaluation	Distance model

**Table 4:** Example of model input data.

Rural	Industrial	Marine	Precipitation	T_annual	RH_annual	TOW
0	1	0	2	11.98	72.1	3218

**Table 5:** Example of results for the case study.

Corr_min	Corr_avg	Corr_max	Range Given by the Model	Category	Range Given by ISO Standard
1.22	1.578	1.91	1.22–1.91	C3 100%	0.7–2.1

**Table 6:** Results obtained by Newton’s method for optimised *b* coefficient.

Corrosivity Category	Value
C2–C3	0.816
C4–C5	0.704

**Table 7:** Results of the example case, using the distance model.

Variable	Example	Result 1	Result 2	Result 3
Location	Dortmund	Bergisch Gladbach	Saint Denis	London
Quality	-	98.10%	98.00%	86.60%
Rural/Urban	Urban	Urban	Urban	Urban
Industrial	Yes	Yes	Yes	Yes
Marine	No	No	No	No
Precipitation	2	2	2	2
T_annual	11.98	11.8	12.3	12.5
RH_annual	72.1	73	73	74
TOW	3218	3149	3146	4021.3
Corr_Zn (µm/year)	-	1.60	1.48	1.67
Corrosion Category	-	C3	C3	C3

## Data Availability

Data sharing is not applicable to this article.
